# A systemic approach to screening high-throughput RT-qPCR data for a suitable set of reference circulating miRNAs

**DOI:** 10.1186/s12864-020-6530-3

**Published:** 2020-01-31

**Authors:** Konrad Pagacz, Przemyslaw Kucharski, Urszula Smyczynska, Szymon Grabia, Dipanjan Chowdhury, Wojciech Fendler

**Affiliations:** 1 Department of Biostatistics and Translational Medicine, Medical University of Lodz, Lodz, Poland; 2 Postgraduate School of Molecular Medicine, Medical University of Warsaw, Warsaw, Poland; 3 Institute of Applied Computer Science, Lodz University of Technology, Lodz, Poland; 4 Dana-Farber Cancer Institute, Harvard Medical School, Boston, USA

## Abstract

**Background:**

The consensus on how to choose a reference gene for serum or plasma miRNA expression qPCR studies has not been reached and none of the potential candidates have yet been convincingly validated. We proposed a new in silico approach of finding a suitable reference for human, circulating miRNAs and identified a new set of endogenous reference miRNA based on miRNA profiling experiments from Gene Expression Omnibus. We used 3 known normalization algorithms (NormFinder, BestKeeper, GeNorm) to calculate a new normalization score. We searched for a universal set of endogenous miRNAs and validated our findings on 2 new datasets using our approach.

**Results:**

We discovered and validated a set of 13 miRNAs (miR-222, miR-92a, miR-27a, miR-17, miR-24, miR-320a, miR-25, miR-126, miR-19b, miR-199a-3p, miR-30b, miR-30c, miR-374a) that can be used to create a reliable reference combination of 3 miRNAs. We showed that on average the mean of 3 miRNAs (*p* = 0.0002) and 2 miRNAs (*p* = 0.0031) were a better reference than single miRNA. The arithmetic means of 3 miRNAs: miR-24, miR-222 and miR-27a was shown to be the most stable combination of 3 miRNAs in validation sets.

**Conclusions:**

No single miRNA was suitable as a universal reference in serum miRNA qPCR profiling, but it was possible to designate a set of miRNAs, which consistently contributed to most stable combinations.

## Background

Molecular genetics has been a major field of study in medicine and physiology since the first successful deoxyribonucleic acid (DNA) isolation as a genetic material and conception of the correct structural model of the DNA [1, 2]. Further work, beginning with the isolation of DNA polymerase I, laid the groundwork for molecular methods of quantifying gene expression [3]. Gene expression is the most fundamental level at which genes drive the phenotype, therefore its measurement remained crucial for not only genetic studies, but also any proteomics or metabolomics research. The need for a fast and reliable way of quantifying the number of copies of a specific gene’s mRNAs gave rise to real-time quantitative polymerase chain reaction (qPCR), which since 1993 arguably became the “golden standard” of gene expression quantification and still continues to be one of the most popular techniques despite the advent of the high-throughput counterparts such as next generation sequencing or hybridization microarrays [4]. In medicine, qPCR, was at first used to detect pathogens’ genetic material and ribonucleic acid (RNA) molecules, among them mRNA and miRNA [5, 6].

MiRNAs represent a group of small non-coding RNA molecules consisting of usually 18–26 nucleotides. They regulate gene expression in a sequence-specific post-transcriptional manner and their expression is often altered in diseases and pathological conditions [7, 8].

A major breakthrough in the field of miRNA studies was the observation that they are stably expressed in human serum, and plasma and as such are good candidates for biomarkers of pathological conditions [9, 10]. Such studies typically use a high-throughput method to screen for candidate miRNAs which are subsequently validated by RT-qPCR [11]. However, qPCR results are highly dependent on parameters of the reaction and varying specificity of probes – settings unique to each experiment. This makes miRNA expression values difficult to directly compare between different qPCR experiments and a wrong choice of a reference can lead to inaccurate and biased conclusions [12–14]. To further complicate matters, qPCR measures the relative abundance of a specific miRNA in the context of a reference gene (normalizer). Therefore, qPCR accuracy relies on both technical conditions and the assumption of an unaltered and stable expression of the internal reference gene. Such a normalizer should be universally abundant in all samples of the material that is investigated and be unaffected by a variety of pathological conditions. Such normalizers have been identified at tissue level and successfully used in multiple studies on mRNA and miRNA quantification alike (ACTB, GAPDH, U6) [15–17]. In biofluids however, the ideal, a single universal reference gene does not exist, and researchers often choose the normalizer for a specific experiment making it difficult if not impossible to pool the results with other studies or perform meaningful meta-analyses. Therefore, the choice of the reference is a crucial and essential step in every qPCR analysis and should be validated on the data acquired in different conditions.

The consensus on how to choose a reference genes for serum or plasma miRNA expression qPCR studies has not been reached and none of the potential candidates have yet been convincingly validated [18, 19]. The most common protocols of normalization involve finding the most stable endogenous reference on an ad hoc, study-specific basis, focusing on normalizers efficient in specific diseases [20–24]; normalization to small-nucleolar RNAs (snoRNAs) such as RNU6B [25, 26] or, when qPCR arrays are used, normalization to mean expression of all miRNAs [27]. The latter approach may only be applied when an array of multiple miRNAs is used, making it unsuitable for validation studies of specific miRNAs or panels; the other approaches hinder the potential of comparing results between studies or rely on different RNA classes which may vary from miRNAs in terms of stability, dynamic range and amplification efficiency [28–30].

Thus, the hunt for the internal reference gene or a set of reference genes adequate for qPCR analysis of serum miRNAs continues. In this article, we proposed a new design of reference gene selection – employing four different methods of measuring expression stability, we created a framework for identification of reference miRNA sets of a variable number of elements– and tested it on all currently available datasets on Gene Expression Omnibus (GEO) platform to find the optimal set of human serum reference miRNA genes.

## Results

### Dataset characterisation and miRNAs filtering

We characterised the included datasets in the Additional file 1: Table S1 [31–37].

### Implementation validation

Bland-Altman analysis and Pearson’s correlation (*r* = 1.0000) showed that our implementations of both versions of NormFinder stood in the excellent agreement with the original. The analysis of the raw data provided in the original GeNorm publication using MetaMirs indicated that our implementation mirrored the results obtained from the original. There was not publicly available version of BestKeeper algorithms, and any results published in the original publication, so we couldn’t perform the validation for our BestKeeper implementation.

### Single miRNA analysis

**Fig. 1:**
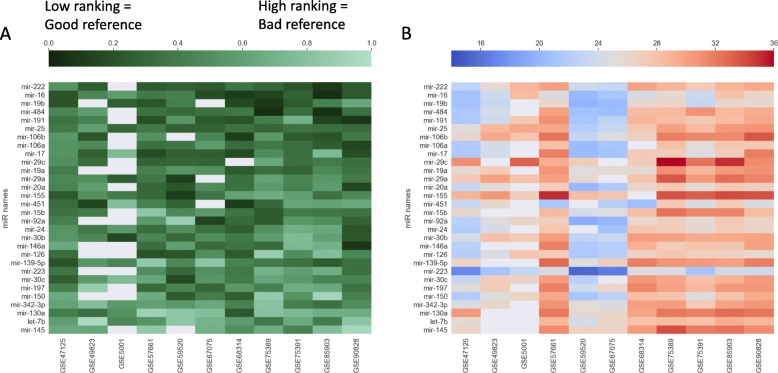
**a** A heatmap of ranking values for the top 30 single miRNA references identified by averaging ranking across datasets. The miRNA shown have the lowest ranking value averaged from all datasets. Color intensity represents the ranking value in a dataset, averaged from the four stability measurement algorithms. The lower the stability value, the better the reference miRNA. MiRNAs at the top were considered the best single normalizers. MiRNAs with missing expression values in more than 20% of datasets were filtered out. Values were not standardized. **b** A heatmap of average raw expression values of miRNAs in each dataset. It suggests that raw expression values of top reference single miRNAs are heterogeneous, thus implying that a combination of them might be a good reference. Expression values were not standardized

We found out that mean rankings, calculated from all sets, of miR-222, miR-16 and miR-19b were the lowest. We performed single miRNA analysis and aggregated the results by averaging their rankings from all datasets. This suggested that those three were the best universal, single miRNA references after selecting miRNAs that were present in more than 80% of the datasets (Fig. 1a). The heatmap of raw expression values showed great heterogeneity of expression amongst the best reference single miRNAs (Fig. 1b). We thus concluded that finding a single best normalization gene would be impossible, as not a single one miRNA achieved the lowest normalization scores in all datasets (see Additional file 1).

### Comparison of rankings between single, combinations of two and three miRNAs

**Fig. 2:**
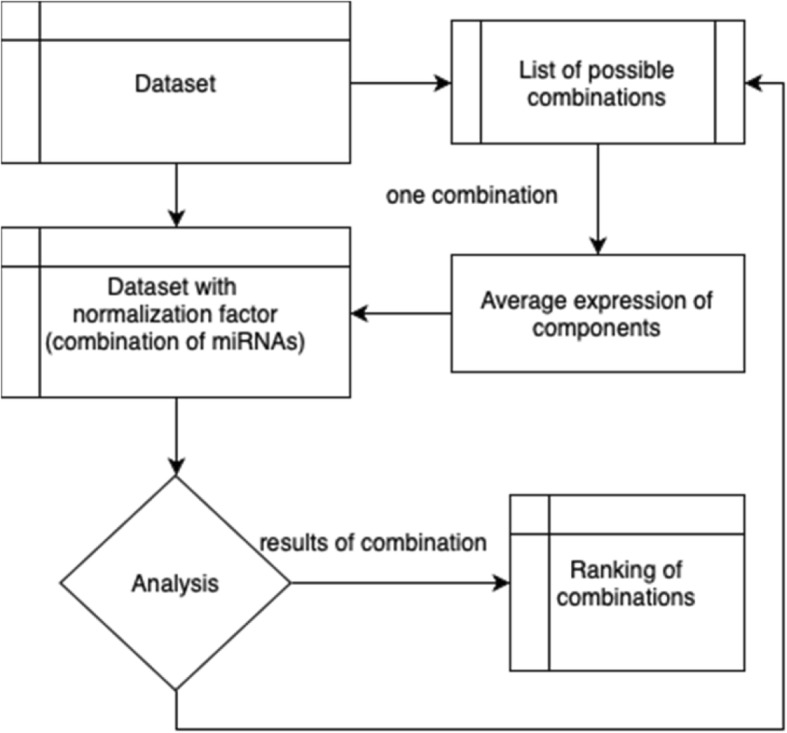
Method of analyzing the stability of miRNA combinations. We decided to analyze combinations of miRNA from a dataset in a context of a dataset. For all possible combinations of miRNAs from a dataset, we sequentially appended an average of expressions of component miRNAs to a dataset (each sample had an additional entry with an average of expression of component miRNAs). Next step was to run the analysis in the same manner as for single miRNAs (as in Fig. 1b), which allowed to identify the average ranking value of a combination in a dataset. Then we removed the combination from the dataset and added another one to ensure that only one combination was present in the dataset at all time. This approach allowed us to aggregate the results from single and combinations of miRNAs without disrupting the workings of the stability measurement tools

**Fig. 3:**
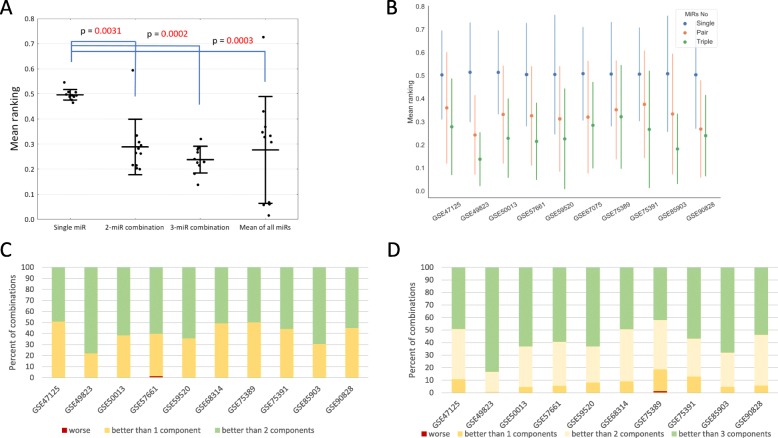
**a** Figure represents the mean and standard deviation of the average ranking of single miRNAs and combinations of 2 and 3 miRNAs as well as mean of all miRNAs in each dataset. Each dot represents the average ranking in a single dataset. *P* values in post-hoc testing > = 0.05 were not shown in the figure. Lower mean ranking represents higher stability. **b** Figure represents the mean and standard deviation of rankings of single miRNAs and combinations of 2 and 3 miRNAs in each dataset. The lower the mean ranking the more suitable the reference candidate. **c** Figure represents the percent of 2-miRNA combinations that were less stable than all of their component miRNAs (red), were more stable than 1 component miRNA (yellow) and better than all of their component miRNAs (green). **d** Figure represents the percent of 3-miRNA combinations that were less stable than all of their component miRNAs (red), were more stable than 1 component miRNA (yellow), were more stable than 2 components (light yellow) and better than all of their component miRNAs (green)

We compared the mean rankings of all combinations of two and three miRNAs as well as mean expression of all miRNAs using the schema shown in Fig. 2. Kruskal-Wallis testing showed *p* < 0.0001 for the comparison (Fig. 3a). In the post-hoc analysis statistically significant were the comparisons between single miRNA and two miRNAs combinations (*p* = 0.0210), three miRNAs combinations (*p* < 0.0001) and mean expression of all miRNAs (*p* = 0.0025), however the difference in the mean ranking was not significant between two and three miRNAs combinations (*p* = 0.2861). Dividing the data into datasets, it was clear that triples of miRNAs proved to be on average the best normalization factors in all datasets occupying the 1st place in rankings in all datasets (Fig. 3b). We also noted that the mean of 2 and 3 miRNAs was on average a better reference gene than its component single miRNAs (Fig. 3c, d), but in around 50% of cases at least one of the component miRNAs was a better reference than the combination. Therefore, we concluded that combinations of three miRNAs proved to be the best normalization factors in all four algorithms.

### Choice of the set of reference miRNAs

Data showed that it was impossible to find a universal single miRNA or a 2- or 3-miRNA combination, which could be reliably used in all 11 datasets as a reference gene. This was partly due to the fact that the overlap of the miRNAs’ presence in the datasets was poor (see Additional file 1). However, we found out there were miRNAs that consistently created part of the top 10 reference combinations of 2 and 3 miRNAs, mainly miR-222, miR-17, miR-320a and miR-27a (see Additional file 1).

We have chosen a set of 13 reference miRNAs: miR-222, miR-92a, miR-27a, miR-17, miR-24, miR-320a, miR-25, miR-126, miR-19b, miR-199a, miR-30b, miR-30c, miR-374. According to our pipeline, we first analyzed the 11 dataset rankings of combinations of 2 miRNAs, specifically combinations that placed first in each ranking. We found out there were multiple combinations placed first in each dataset. This was possible, because our algorithm evaluated one combination at a time in the context of an original dataset. After assessing possible sets of reference miRNAs in the validation step on the dataset rankings of combinations of 3 miRNAs, we proposed a set with the lowest normalization score and with possibly minimal known dynamic range in serum. By deriving combinations of 3 miRNAs our chosen dataset covered all first positions in the 11 dataset rankings both for 2 and 3 miRNA combinations.

**Fig. 4:**
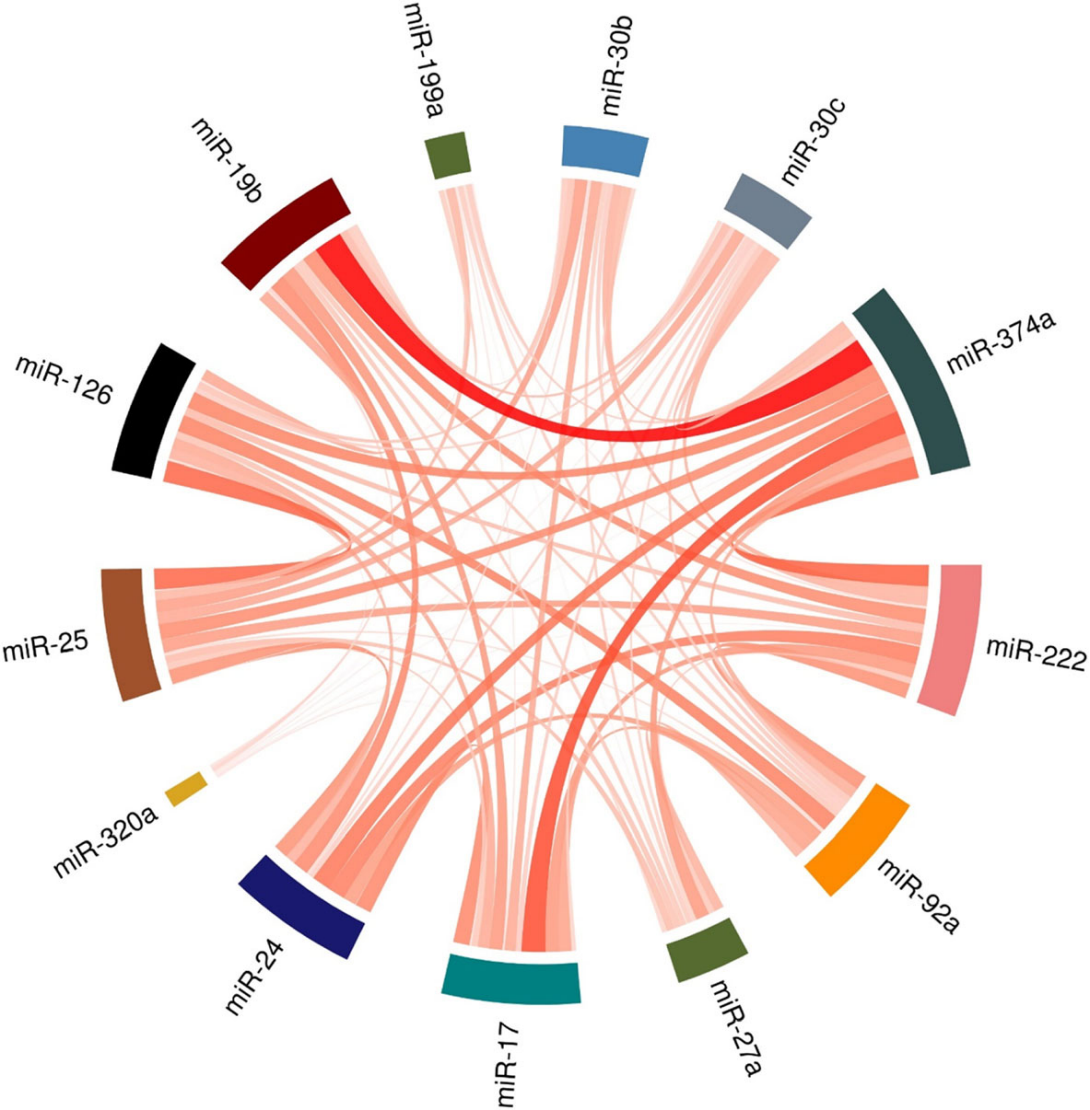
We counted the number of times two miRNAs occurred in all combinations of 3 miRNAs, which placed 1st in the 11 dataset rankings. We divided each singular count by the number of combinations in a dataset containing the counted combination and summed the counts from all occurrences of a pair. miR-374a, miR-222, miR-25, miR-126, miR-24 had the highest contribution to creation of the best normalizing combinations of 3 miRNAs

Pairwise analysis of miRNAs from the 11 datasets showed the strongest affinity between: miR-374a and miR-19b, between miR-374a and miR-17, and weaker affinity between miR-25 and miR-126 (Fig. 4). miR-374a, miR-222, miR-25 and miR-126 had the highest contribution to creating the most stable combinations of 3 miRNAs (Fig. 4).

### External validation of the chosen set of miRNAs

**Fig. 5:**
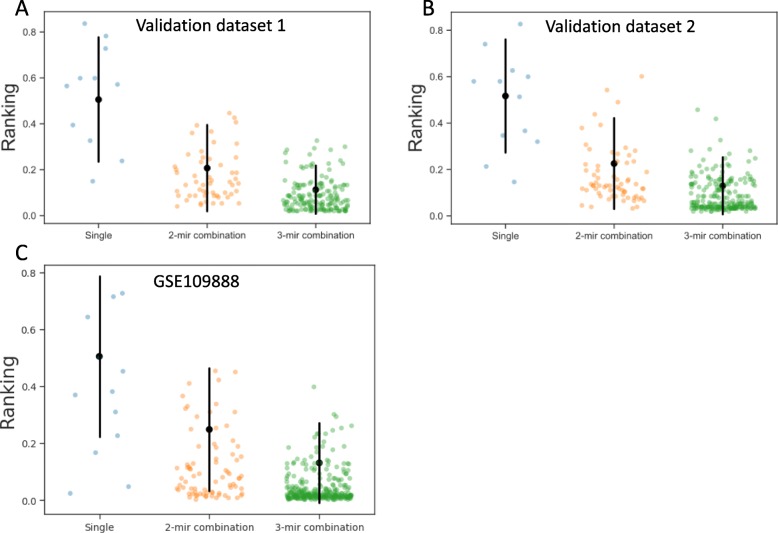
The mean and the standard deviation of ranking of all normalizing factors in two unpublished validation sets - panels **a** and **b** - and a publicly available dataset GSE109888 - panel **c** (black point and lines; description of the validation datasets experiments in the Additional files 1, 2, 3, 4 and 5). Colored dots represent ranking values of combinations of miRNAs from our chosen set. Our candidate normalization factors clustered towards the lower values of ranking (better stability)

**Fig. 6:**
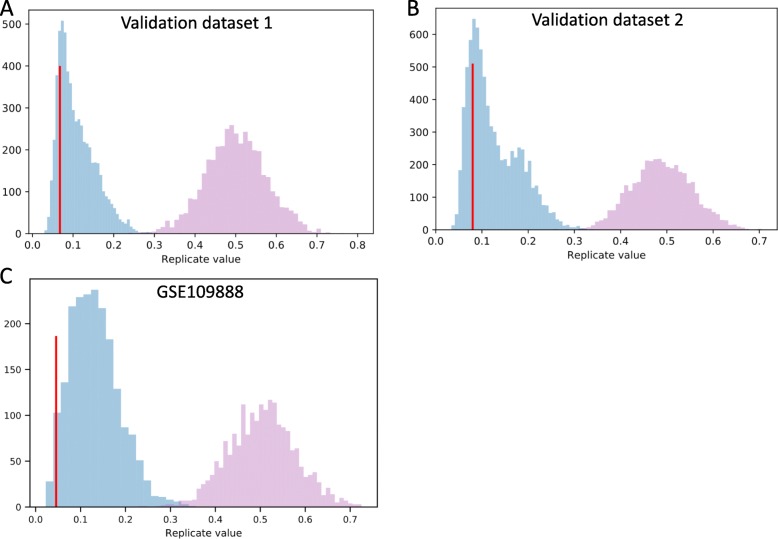
We performed the validation of the chosen set of 13 miRNAs as suitable reference genes. Figures represent histograms of distributions of mean ranking of randomly selected 13 miRNAs (blue). Panels **a** and **b** show two validation sets attached in the Additional files and panel **c** shows data from a publicly available GSE109888. We sampled 13 random ones from the pool of miRNAs presented in a validation dataset 2000 times creating 2000 replicates of mean ranking of derived 3 miRNA combinations. This allowed to plot empirical distribution of mean ranking of combinations derived from any arbitrarily selected 13 miRNAs. Shown are mean rankings of single miRNAs (pink) and combinations of 3 miRNAs (blue). A red vertical line marks mean ranking of 3 miRNA combinations derived from the chosen set. The lower the average ranking the more suitable the combination to be a reference gene. Average ranking of combinations derived from the chosen set (the red vertical lines) was lower than 83.32, 84.76 and 97.45% of all average rankings in three validation sets, respectively. In summary, combination of 3 miRNAs picked from our set of 13 were repeatedly within top 15% of best normalizers in two datasets and significantly outperformed single-miRNA normalizers

We validated the set of 13 reference miRNAs on three external qPCR datasets – two unpublished datasets from patients with head and neck tumors and one publicly available dataset from a study including patients with rheumatoid arthritis [38] – see Additional files 2, 3 and 4. Figure 5 represents the results of the external validation. Rankings of the combinations of the chosen miRNAs clustered towards lower ranking. Validation data confirmed that combinations of two and three miRNAs were a better reference than a single miRNA. We also identified that our chosen set showed low mean ranking of derived three-miRNA combinations in the overall distribution of mean ranking of combinations derived from random 13 miRNAs (Fig. 6). Average ranking of combinations derived from the chosen set was lower than 83.32, 84.76 and 97.45% of all average rankings in three validation sets, respectively. This positive control indicated that our choice of a set created more stable references than any random 13 miRNAs, which validated our approach to selecting the set. We found out that for three external datasets the best combination of 2 chosen miRNAs placed 3rd in the combined rankings and multiple combinations of 3 chosen miRNAs placed 1st in the combined rankings (Table 1). miR-24, miR-222 and miR-27a constituted the combination with the lowest average ranking in validation analysis, among combinations of 3 miRNAs present in all two validation datasets (Additional file 1: Table S2). Detailed rankings of combinations derived from the chosen set and the best combinations in validation sets are located in the Additional files 1: Table S3 and distribution of mean rankings of combinations of 2 miRNAs in comparison with the mean of our chosen set is in the Additional file 5. As such we concluded that our normalization scheme is a valid tool for normalizing serum miRNA qPCR data and the proposed set of 13 miRNAs, emphasizing one combination of 3 miRNAs (miR-24, miR-222 and miR-27a), can be used as a viable reference for such experiments .

**Table 1:** Performance of the chosen miRNAs in the validation datasets

Validation dataset	Place of the best single miRNA	Place of the best pair	Place of the best triple
1	6	3	1
2	4	3	1
GSE109888	1	1	1

We validated our chosen set on two qPCR datasets. In each dataset we used our algorithms to calculate normalization rankings for all possible combinations of miRNAs present in a dataset and then noted the highest place in the rankings of the combination derived from our chosen set. Full list in the Additional file 1: Table S3. The combination with the lowest average (0.0231) ranking from the validation datasets consisted of miR-24, miR-222 and miR-27

## Discussion

Our study shows that combinations of two or preferably three miRNAs make for a better reference than single miRNAs across a variety of clinical conditions and experimental setup. While it is difficult to pinpoint a single best combination of miRNAs that can be used in all situations, a set composed of miRNAs chosen from among: miR-222, miR-92a, miR-27a, miR-17, miR-24, miR-320a, miR-25, miR-126, miR-19b, miR-199a, miR-30b, miR-30c, miR-374a seems to be a safe, conservative choice that can be readily adopted as a standard for circulating miRNA biomarker studies.

We proposed a set of miRNAs that we validated on new data to show that only 13 miRNAs were needed to be included in an analysis to acquire a stable endogenous normalization factor. We propose to normalize qPCR data to the combination of 3 miRNAs, which have the lowest normalization score, equivalent to the lowest ranking, using our algorithm pipeline and deriving combinations from the set of 13 proposed miRNAs. Our approach found a good reference in a systemic way taking into the account variety of qPCR datasets. The inclusion of datasets with different patients’ conditions and treatments ensured that our results could be generalized as much as possible and the impact of different conditions of experiments on the choice was minimized.

Using spike-in reference has emerged as a trend and in fact has been used in many cases, but is not without specific drawbacks, all of which limit its applicability in biofluid studies. Spike-in methods operate on two assumptions: 1) the same amount of spike-in RNA is added to each sample; 2) synthetic spike-in transcripts behave in the same way as endogenous transcripts. It has been shown that both of those assumptions are often false and consequently disrupt the results [39, 40]. This is due to the inherent biological variability of sample storage, quality, degree of degradation and potential confounding factors. Therefore, a known-concentration spike-in may produce erroneously globally increased or decreased expression level of all evaluated miRNAs. While in experimental conditions such as cell cultures or isogenic animals, between-sample variability is largely reduced by the methodological constraints, in the clinical setting an endogenous standard is thus a far more safe point of reference as even in a degraded sample the miRNA/reference ratio should remain largely the same if both are affected by the physical, biological and chemical factors similarly. Given that our proposed references also members of the miRNA family both the investigated ones and the reference ones should maintain their relative ratio indicative for the investigated pathological condition even across samples of varied quality. Additionally, there has been no consensus on the amount of the spike-in control added to the sample, which still leads to inter-experiment bias, while any endogenous reference potentially services more than one experiment.

The biggest obstacle to overcome in the study were long computational times. The need to calculate normalization scores for each new combination was time-consuming. Even though a single combination did not take long to calculate (time below 1 s), the sheer number of combinations going as high as 10^7^ made our whole analysis take hours in the case of 2 miRNAs combination to days in the case of 3 miRNAs combinations. We explored other avenues of tackling the issue of long computation time by reducing the number of miRNAs included in the creation of combinations. We checked whether the best reference single miRNAs could be combined into the best reference combinations of 2 and 3 miRNAs. We showed that such an approach did not guarantee that the combinations would be a good reference, since some combinations created from miRNAs were worse than their component miRNAs.

More miRNAs in the combination did not translate to a strictly better reference combination. We carried out the analysis only for combinations of 2 and 3 miRNAs, because longer combinations would require computational times of months. The maximum number of miRNAs that can be included in the combination is equal to the number of miRNAs in the dataset. Such a combination would be equal or at least non-inferior to normalizing to the mean of expression of all miRNAs and we showed that this reference was not a reliable one and should not be used. Also, combinations of 3 miRNAs did not differ statistically significantly from combinations of 2 miRNAs, despite the pronounced difference in the mean rankings. We hypothesize there is a threshold number of miRNAs, after which the stability reaches a plateau and then starts to decline. Drobna et al. measured normalization scores of only NormFinder algorithm for different number of miRNAs in a combination. Their data indicated that the plateau was quickly reached around the number of 3–4 miRNAs [24], which strengthens our belief.

Finding the suitable reference gene for qPCR analysis of human serum miRNAs has never seemed more relevant than now. The number of projects that use circulating miRNAs as biomarkers is increasing and the need to find a good reference was never direr, since the choice of the reference is crucial for the interpretation of the results and wrong choice can threaten the accuracy of the results. Finding the universal single or even a small group of reference miRNAs for human serum miRNA gene expression analysis by qPCR seemed to be impossible based on our results and this agreed with the work of others [18, 20, 41]. The idea to use multiple algorithms to find a reference gene was previously described [18, 24, 42]. In short, Marabita et al. described a new normalization algorithm using three different normalization tools and presented case-study applications on single datasets. Mallona et al. defined an approach using 4 normalization algorithms to create a unified normalization score by calculation of a footrule distance matrix and finding a consensus ranking by Monte Carlo cross entropy algorithm. They also used only single study approach to measure stability of genes in plants. Drobna et al. introduced a normalization pipeline that included 4 different normalization algorithms, which they applied to several datasets of patients with acute lymphoblastic leukemia. They also decided to use a combination of 3 miRNAs as a reference based on the normalization scores of single miRNAs. All the studies above included a step of literature-based arbitrary preselection of candidate miRNAs.

The aforementioned approaches have several areas, which we improved in our work. First of all, we showed that choice of miRNA reference should not be made based on a single qPCR study, because no single good reference miRNA was reproducible in all experiments. Moreover, we proved that the mean expression of a combination of 2 or 3 miRNAs was a better reference than the expression of a single miRNA. In that regard our analysis mirrored the conclusions already made before by others [43, 44].

In order to determine the potential factors that would impair the performance of miRNAs included in our normalizer set we performed a literature search of biological significance of the chosen miRNAs. Due to large number of pathological conditions that potentially impact the levels of circulating miRNAs, we compiled a list of conditions which had been evidenced to significantly alter expression levels of the corresponding miRNAs from the proposed, reference set (Table 2). This should allow for an informed decision about what miRNAs to include in a reference panel depending on known pathological conditions in a studied population. Moreover, we summarized the data about previous usage of aforementioned miRNAs as reference miRNAs in paragraphs below. Curiously, miR-222 has already been established as a serum reference miRNA in patients with pleural effusion and in the study of estrogen-responsive miRNAs associated with acquired protein S deficiency in pregnancy [41, 53]. Combination of 5S-rRNA and miR-92a enhanced the normalization quality compared to using only 5S-rRNA in the study of optimal small-molecular reference RNA for body fluid identification [54]. miR-27a was found to be stably expressed in rectal cancer tissue, but the downregulation of its exosomal expression has been associated with amyotrophic lateral sclerosis [55, 56]. miR-17 was found to be overexpressed in many human cancer tissues and to promote cell growth. miR-17 is a member of miR-17-92 cluster, which had been termed onco-miR-1 and its overexpression was proposed to be an early non-specific sign of cancer [57]. miR-24 was notably a worst reference in a cardiovascular diseases’ study involving 7 small non-coding RNAs (miR-16, SNOU6, 5S, miR-19b, miR-24, miR-15b, let-7i), although the authors of the study employed only one normalization algorithm – BestKeeper on top of comparative delta Ct analysis [58]. Serum expression of miR-320a was not previously considered as a reference miRNA, but was connected to several conditions including: metabolic syndrome, epithelial ovarian cancer and inflammatory bowel disease [59, 60]. miR-25 had been previously confirmed as a suitable circulating reference gene [20, 24, 41]. Expression of circulating miR-126 was found to be associated with disease free survival in patients with squamous cell lung cancer [45]. It was also implicated in the suppression, migration and invasion of non-small-cell lung cancer cells via targeting CCR1 as well as other molecular functions, but it was not considered as a suitable reference gene [46, 47]. miR-19b had been found out as a decent reference miRNA in the previously mentioned evaluation of 7 potential normalizers in studies focused on cardiovascular diseases [58]. The findings of Zuberi et al. suggested that miR-199a downregulation might be a potential indicator for progression of epithelial ovarian cancer [48]. This miRNA has not been previously viewed as a potential reference miRNA. miR-30b was previously considered as a reference miRNA in self-collected cervicovaginal tissue specimens in a study that evaluated 11 reference candidate small non-coding RNAs [49]. miR-30c had been used before as a biomarker for detection of autologous blood transfusions and as an early predictor of recurrence of localized stage I non-small cell lung cancer after surgical resection [50, 51]. The search of PubMed publication database turned out 3 articles about miR-374a and none suggested specific profiles that included miR-374a nor its suitability as a reference miRNA.

**Table 2:** List of exclusions from consideration as a reference miRNA based on the known, experimentally validated connections

microRNA	Reports on differential expression in serum or plasma due to specific clinical conditions	Reference
hsa-miR-222	Gastric cancer	[45]
hsa-miR-92a	Acute myeloid leukemia, acute myocardial infarction, potential marker of atherosclerosis, metabolic syndrome, bladder cancer, systemic lupus erythematosus	[46–49]
hsa-miR-27a	Acute pulmonary embolism, colorectal cancer	[52, 61]
hsa-miR-17	Elevated in many types of cancer, acute ischemic stroke, endometriosis, acute myocardial infarction	[62–64]
hsa-miR-24	Coronary heart disease, type 2 diabetes mellitus, early breast cancer	[65, 66]
hsa-miR-320a	Esophageal adenocarcinoma, Barret’s esophagus, arrhythmogenic cardiomyopathy	[67, 68]
hsa-miR-25	Osteosarcoma, breast cancer, papillary thyroid carcinoma, pancreatic cancer, gastric cancer, hepatocellular carcinoma, esophageal adenocarcinoma	[69–74]
hsa-miR-126	Non-small-cell lung cancer, chronic heart failure, acute myocardial infarction, ischemic stroke, type 2 diabetes mellitus	[64, 75–78]
hsa-miR-19b	Knee osteoarthritis, diabetic cardiomyopathy, acute myocardial infarction, gastric cancer, prostate cancer, lung cancer	[79–84]
hsa-miR-199a	Glioma, hepatocellular carcinoma, acute myocardial infarction, colorectal cancer, osteosarcoma	[85–89]
hsa-miR-30b	Active tuberculosis	[90]
hsa-miR-30c	HLTV-1 infection, Duchenne muscular dystrophy, active pulmonary tuberculosis	[91–93]
hsa-miR-374	Acute Graft-versus-Host disease	[94]

We have performed a literature search in MEDLINE and PMC databases (through www.ncbi.mln.nih.gov/pubmed portal) looking for published evidence of pathological conditions influencing levels of circulating miRNAs included in the proposed reference set. Only human and experimentally validated studies were included, and the expression of a miRNA must have differed significantly between study and control groups

## Conclusions

Our work showed that using single miRNAs as references in biofluids provides a significantly worse reference for qPCR than a combination of two or three miRNAs. We also showed that the strategy of normalizing to a combination of miRNAs was more stable and predictable than normalizing to the average of expression of all miRNAs in a dataset. The proposed set of 13 miRNAs that reproducibly contributed to the selection of best normalizer combinations should be validated in further studies on patients with different clinical conditions, nonetheless we have showed that based on our in silico validation the arithmetic mean of 3 miRNAs (miR-24, miR-222 and miR-27a) was sufficiently stable to be used as a reference for serum miRNA qPCR profiling.

## Methods

### Data acquisition

**Fig. 7:**
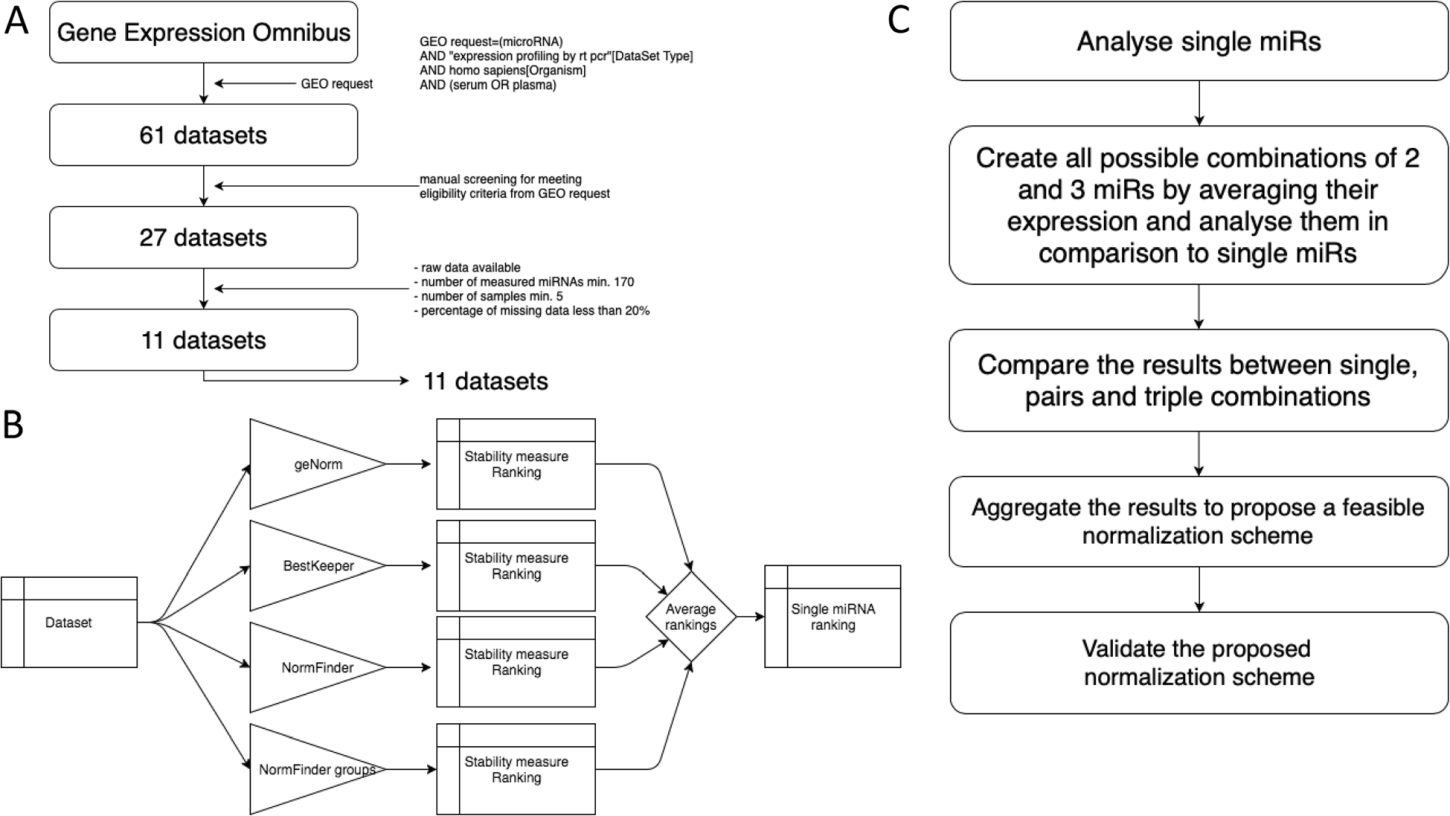
**a** Flowchart of the steps taken in our study to acquire 11 datasets of miRNA expression in serum measured by qPCR and to identify the most suitable single miRNA or a set of miRNAs to use as reference. **b** Flowchart of our approach to analysis of single miRNAs. Each dataset was analyzed by the same four algorithms implemented in the Python programming language. Algorithms independently assigned a stability value to each miRNA. We changed algorithms to assign a ranking from 0 to 1 based on the stability value (the lower the ranking value, the better the reference), thus each miRNA had 4 ranking values. We averaged the four values for each miRNA, which resulted in a single measure of stability and aggregated the results from 11 datasets. **c** The outline of the two-pronged approach of our analysis. We first analyzed all single miRNAs and then created all possible average expressions of two or three miRNA-combinations and analyzed the suitability of single miRNAs and their combinations as a good qPCR reference using the algorithms shown in Fig. 1b

To identify datasets pertaining to biofluid miRNA profiling with qPCR, we carried out a GEO search with a query: ((miRNA) AND “expression profiling by RT PCR”[DataSet Type]) AND *Homo sapiens* [Organism] AND (serum OR plasma), which yielded 61 hits (accession date: 30.10.2017). Afterwards, we extracted datasets, which fulfilled all of the following criteria: include miRNA high-throughput methods, raw data must be published, number of miRNAs measured at least 170 – many studies included only a limited array of miRNAs, often as a validation of several miRNAs previously identified by another measurement method, number of samples at least 5 – normalizing algorithms require it for reliable calculations, percentage of missing data lower than 20% (Fig. 7a). We manually curated each dataset removing duplicates miRNAs and chip-specific control probes. While curating a single dataset, we also removed all miRNAs, which did have missing values in that particular dataset. We treated not achieving a threshold of detection in RT-qPCR as missing values.

All the data used in this study can be accessed via the GEO platform (https://www.ncbi.nlm.nih.gov/geo) using accession link found in the Additional file 1: Table S1.

### Algorithms implementation, normalization scoring, aggregating results from different algorithms

We implemented three algorithms: BestKeeper, NormFinder (in two versions), GeNorm in the Python programming language following formulas extracted from the appropriate original publications [12, 24, 52]. Python serves well for handling large-scale data and extensive calculations, because it provides well-optimized built-in functions and modules tailored to deal with data in tabular form (*pandas* module). BestKeeper proposes a set of housekeeping genes and then introduces the correlation coefficient as a reference. It ranks genes basing on the standard deviation of their expression across samples. Genes with lower standard deviation are considered more stable. It calculates the normalization score for each possible set of housekeeping genes. The NormFinder model-based algorithm is proposed in two variants – for datasets with multiple groups of samples and for datasets with only one group. For a generic algorithm, the inter- and intragroup variations are estimated for each candidate gene. The combined variations are the basis for stability value. The authors state that the proper measure of stability is the distribution in the developed model, but for practical reasons it is reduced to one-dimensional value equal to the sum of mean and standard deviation of the aforementioned distribution. GeNorm ranks reference genes according to stability value M, where lower M means higher stability [12]. The basis for calculation of stability measure for a chosen gene is log2-transformed ratio of expressions between the ranked gene and each of the remaining genes in the dataset. The stability measure M is then calculated as an arithmetic mean of standard deviations of pairwise expression ratios. In the original algorithm, the procedure begins with a set of candidate genes, which is a small subset of all available genes. This approach is proposed due to computational efficiency of the solution. For each candidate gene, stability value M is computed, and the gene with highest M value (i.e. worst stability) is excluded from the dataset. Then, the M-value calculations are repeated for the remaining genes, until there are only two best genes left.

Then we assigned a ranking specific for each algorithm, which varied in an interval [0, 1) and was calculated as follows:

$$ \mathrm{Dataset}\ \mathrm{ranking}=\frac{\mathrm{place}\ \mathrm{in}\ \mathrm{the}\ \mathrm{sorted}\ \mathrm{array}\ \mathrm{of}\ \mathrm{normalization}\ \mathrm{scores}-1}{\mathrm{number}\ \mathrm{of}\ \mathrm{elements}\ \mathrm{in}\ \mathrm{the}\ \mathrm{array}\ } $$

The final normalization score of a miRNA in a dataset was an arithmetic average of four algorithm-specific rankings (Fig. 7b). The final normalization scores of all miRNAs created a normalization ranking. The lower the ranking, the better the normalization factor.

### Implementation validation

We validated the results we received from our implementation of algorithms. For NormFinder, we carried out an analysis of RT-qPCR miRNA expression (GEO ID: GSE47125) with the readily available Microsoft Excel plug-in created by the authors of the original NormFinder article and our implementation of NormFinder. We compared the results using Bland-Altman plot analysis and Pearson’s correlation. There was no readily available, original, open-source and updated to work with current versions of operating systems’ software implementation of GeNorm and BestKeeper. In case of GeNorm we used the raw data provided by the authors of the original manuscript and ran the analysis for the leukocytes dataset checking for the overlap of the order of the genes. The original BestKeeper article did not include any analyzed data and there is no implementation available in the open-access fashion, so we could not validate our implementation of BestKeeper with the original. In the face of the same problem, Marabita et al. decided to implement their own simplified version of the algorithm, but we decided not to use it [18].

### Multiple miRNAs as a single normalization factor

BestKeeper and GeNorm do not allow scoring of multiple miRNAs as a single normalization factor, so we had to devise a way of combining multiple miRNAs. We decided to average expression values of multiple miRNAs (either two or three) by arithmetic mean, thus creating a new entry compatible with the rest of the original dataset. The length of such prepared dataset was larger than the original by one – the new combination of multiple miRNAs. Then we could input the original dataset with the added entry to normalization algorithms, which allowed for evaluation of the combination set in the context of the whole dataset.

Inclusion of all possible combinations of miRNAs into the dataset would distort the image of the measured expression, as the number of all possible combinations would always be vastly greater than that of actual miRNAs quantified. This, in turn, would significantly impact the standard deviation of the expression of miRNAs and hinder the possibility to calculate the normalization scores of NormFinder and GeNorm algorithms. Therefore, we decided to add single combinations sequentially and by doing so minimize the effect of the addition of every new entry. Notably, expanding the dataset by adding all possible combinations would be without consequence for BestKeeper as it does not use standard deviation or its derivatives, but we wanted to create a uniform pipeline for all three algorithms. Therefore, only one combination at a time was present in a dataset during calculations of normalization scores. We analysed all possible combinations of 2 and 3 miRNAs in the aforementioned way (Fig. 7c). We did not attach more than one combination of miRNAs to a dataset simultaneously.

### Combining information from different datasets – finding the normalization set

According to our workflow (Fig. 7c), we created a combined ranking for all normalization factors – single miRNA, combinations of 2 and 3 miRNAs. We algorithmically found a small set of single miRNAs, which could be further used to create combinations of pairs and triple miRNAs selected from the set. We wanted to ensure that the best miRNAs present in the highest-scoring combinations of 2 and 3 miRNAs were included amongst the chosen set of miRNAs and that the set is as small as possible without compromising the stability of combinations created from it.

We focused on the 11 dataset rankings of combinations of 2 miRNAs to find a reliable set of miRNAs and validated their performance on the 11 dataset rankings of combinations of 3 miRNAs. First step was to find the smallest miRNA set that could be used to create combinations of 2 miRNAs, which placed as the best normalization factors in the 11 dataset rankings of combinations of 2 miRNAs. The second step involved validating whether a chosen set could be used to create combinations of 3 miRNAs that also placed first in all the 11 datasets. At last we excluded sets containing miRNAs with a known labile or dynamic expression in serum or plasma.

We performed a pairwise analysis of miRNAs to better illustrate the relationships between them in the chosen set. Therefore, we counted the number of times two miRNAs occurred in all combinations of 3 miRNAs, which placed 1st in the dataset rankings. We divided each singular count by the number of combinations in a dataset containing the counted combination and summed the counts from all occurrences of a pair. This ensured that the counts were not weighted by existence of a one dataset with a multitude of miRNAs. The resulting matrix was used to create a chord diagram illustrating the “normalizing affinity” between pairs of miRNAs from the chosen set.

### External validation of results

We have acquired three serum miRNAs qPCR datasets (description of the datasets in the Additional files 2, 3 and 4). We tested our chosen set of reference miRNAs, which we acquired in the previous step. We took each dataset and ran our normalization algorithms for them and then verified how our chosen miRNAs and combinations derived from them scored and compared with the normalization ranking of the dataset. Additionally, we reported the mean ranking of all combinations derived from the chosen 13. We wanted to know whether our miRNA set was truly better than any random set, therefore we sampled random 13 miRNAs 2000 times and estimated the distribution of the mean of rankings of combinations derived from a set of 13 random miRNAs. We reported the position of the mean of our chosen set in this distribution by providing the percentage of random sets surpassed by our set, which we treated as *p*-value of a Monte-Carlo testing procedure.

### Statistical analysis

Initial pre-processing of the expression data and tidying was performed in Microsoft Excel 2016. We did the Kruskal-Wallis testing for comparisons of rankings between single and combinations of 2 and 3 miRNAs using statistical software package Statistica (13.1 StatSoft). We devised the Monte-Carlo analysis in the Python programming language using module ‘itertools’.

## Supplementary information

**Additional file 1.** Supplementary tables. Contains supplementary tables created by the authors.

**Additional file 2.** Validation dataset 1. Contains results of the qPCR profiling of circulating miRNAs in people with head and neck tumors. Samples are in columns, miRNAs are in verses.

**Additional file 3.** Validation dataset 2. Contains results of the qPCR profiling of circulating miRNAs in people with head and neck tumors. Samples are in columns, miRNAs are in verses.

**Additional file 4.** Validation dataset GSE109888. Publicly available on Gene Expression Omnibus platform under the accession number GSE109888. Contains results of the qPCR profiling of circulating miRNAs in people with rheumatoid arthritis [38]. Accession link: https://www.ncbi.nlm.nih.gov/geo/query/acc.cgi?acc=GSE109888.

**Additional file 5.** Supplementary figures. Contains additional figures not included in the main body of the manuscript: 7 additional figures containing validation of the proper implementation of the three used algorithms and figures from the Monte Carlo simulations on the external validation datasets.

## Data Availability

The 11 Gene Expression Omnibus datasets used in the process of finding miRNA reference genes are available on the GEO platform (https://www.ncbi.nlm.nih.gov/geo/) under appropriate GSE identifiers and accession links (see Additional file 1: Table S1). Validation datasets used in this study were attached as Additional files to this article. One of them is publicly available in the GEO database (https://www.ncbi.nlm.nih.gov/geo/query/acc.cgi?acc=GSE109888).

## Abbreviations

DNA: Deoxyribonucleic acid
GEO: Gene expression omnibus
RNA: Ribonucleic acid
rt-qPCR: real time quantitative polymerase chain reaction
snoRNA: small-nucleolar RNA
