# Supplementary material for: A systemic approach to screening high-throughput RT-qPCR data for a suitable set of reference circulating miRNAs
Source: BMC Genomics. 2020 Jan 31;21:111. doi: 10.1186/s12864-020-6530-3 (PMC6995162; doi:10.1186/s12864-020-6530-3)
Supplement: Supplementary file 5 — Additional file 5. Supplementary figures. Contains additional figures not included in the main body of the manuscript: 7 additional figures containing validation of the proper implementation of the three used algorithms and figures from the Monte Carlo simulations on the external validation datasets. [file 12864_2020_6530_MOESM5_ESM.pptx]

## Slide 1
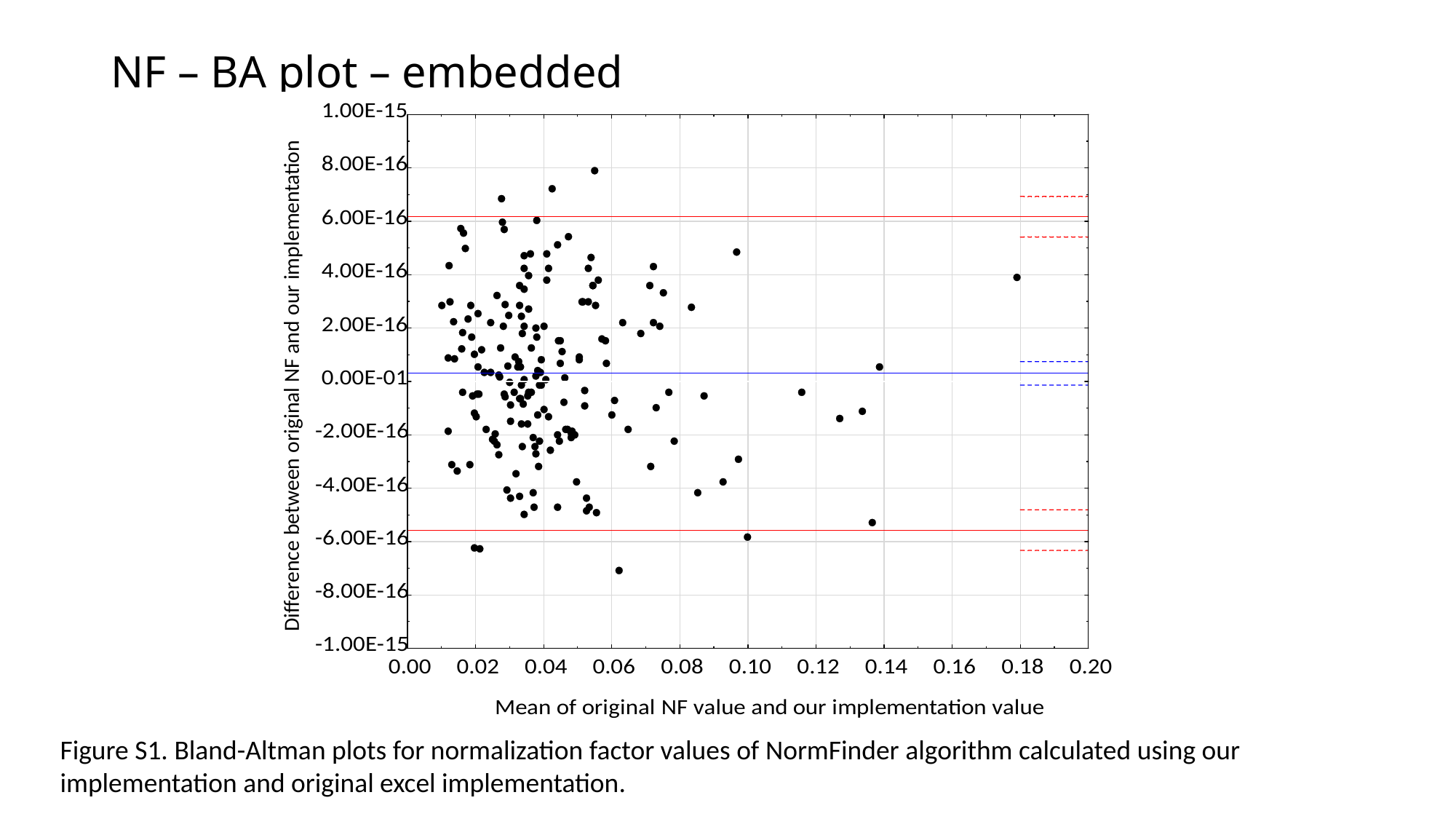

# NF – BA plot – embedded
Figure S1. Bland-Altman plots for normalization factor values of NormFinder algorithm calculated using our implementation and original excel implementation.

## Slide 2
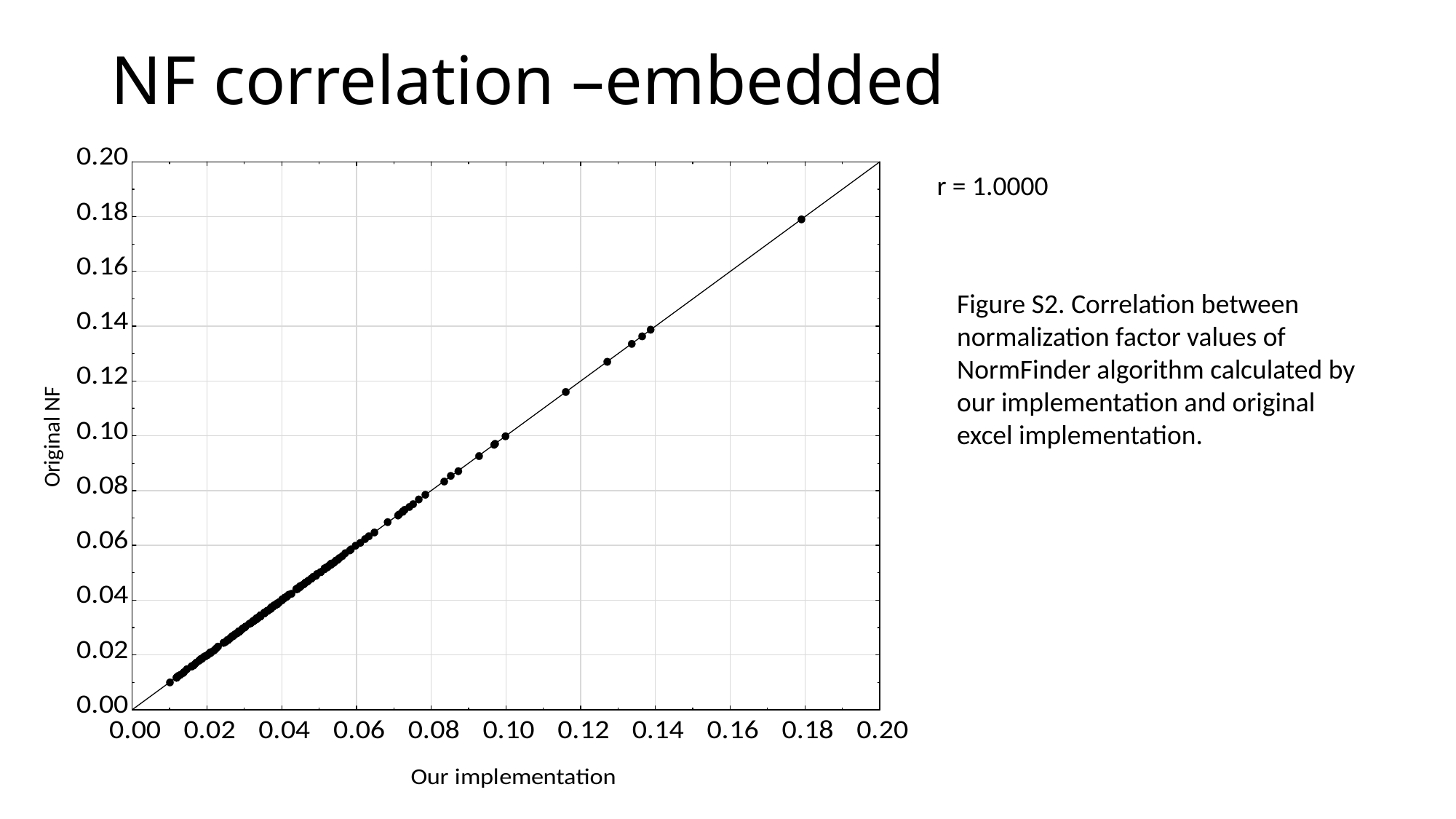

# NF correlation –embedded
r = 1.0000
Figure S2. Correlation between normalization factor values of NormFinder algorithm calculated by our implementation and original excel implementation.

## Slide 3
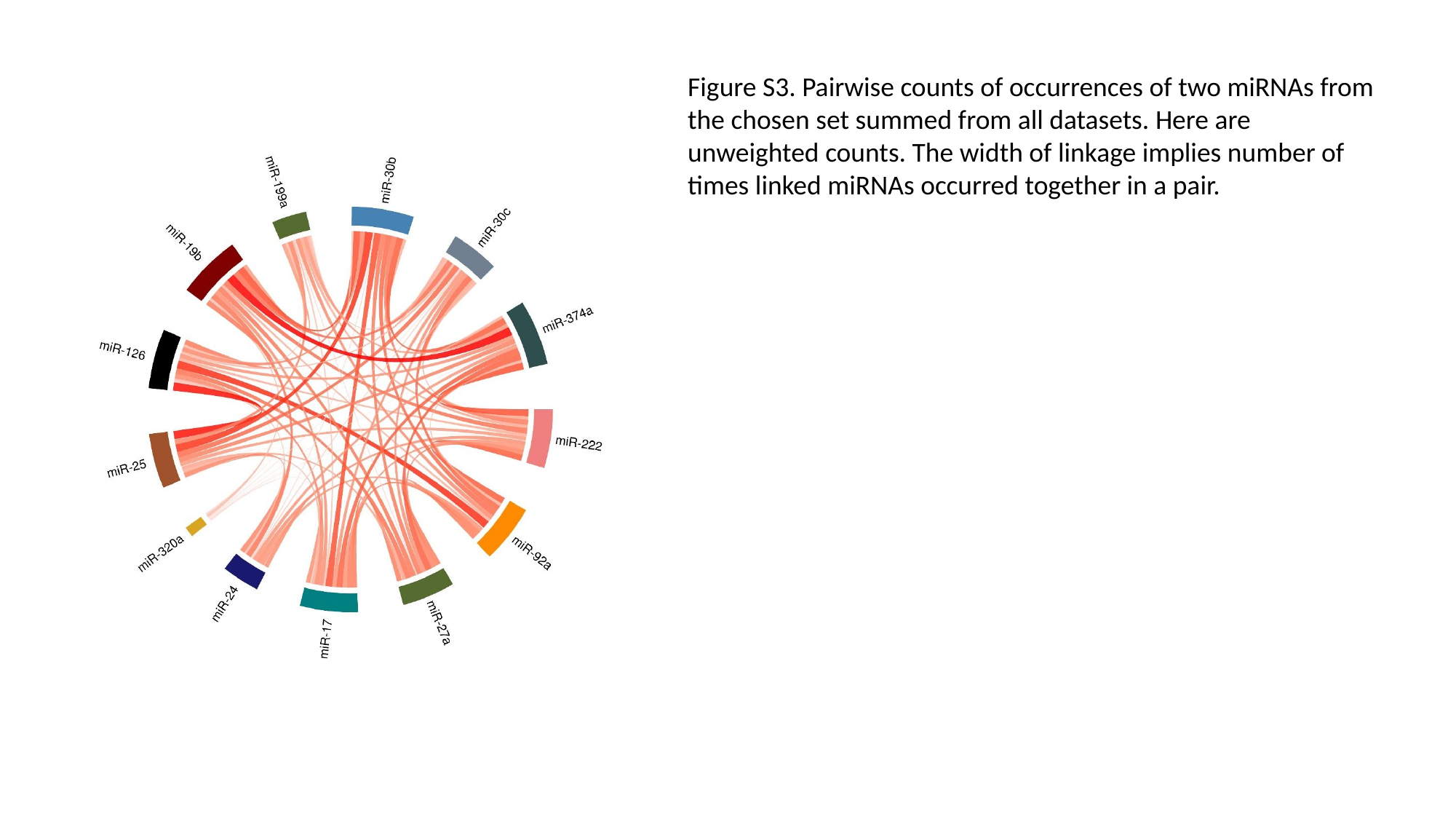

Figure S3. Pairwise counts of occurrences of two miRNAs from the chosen set summed from all datasets. Here are unweighted counts. The width of linkage implies number of times linked miRNAs occurred together in a pair.

## Slide 4
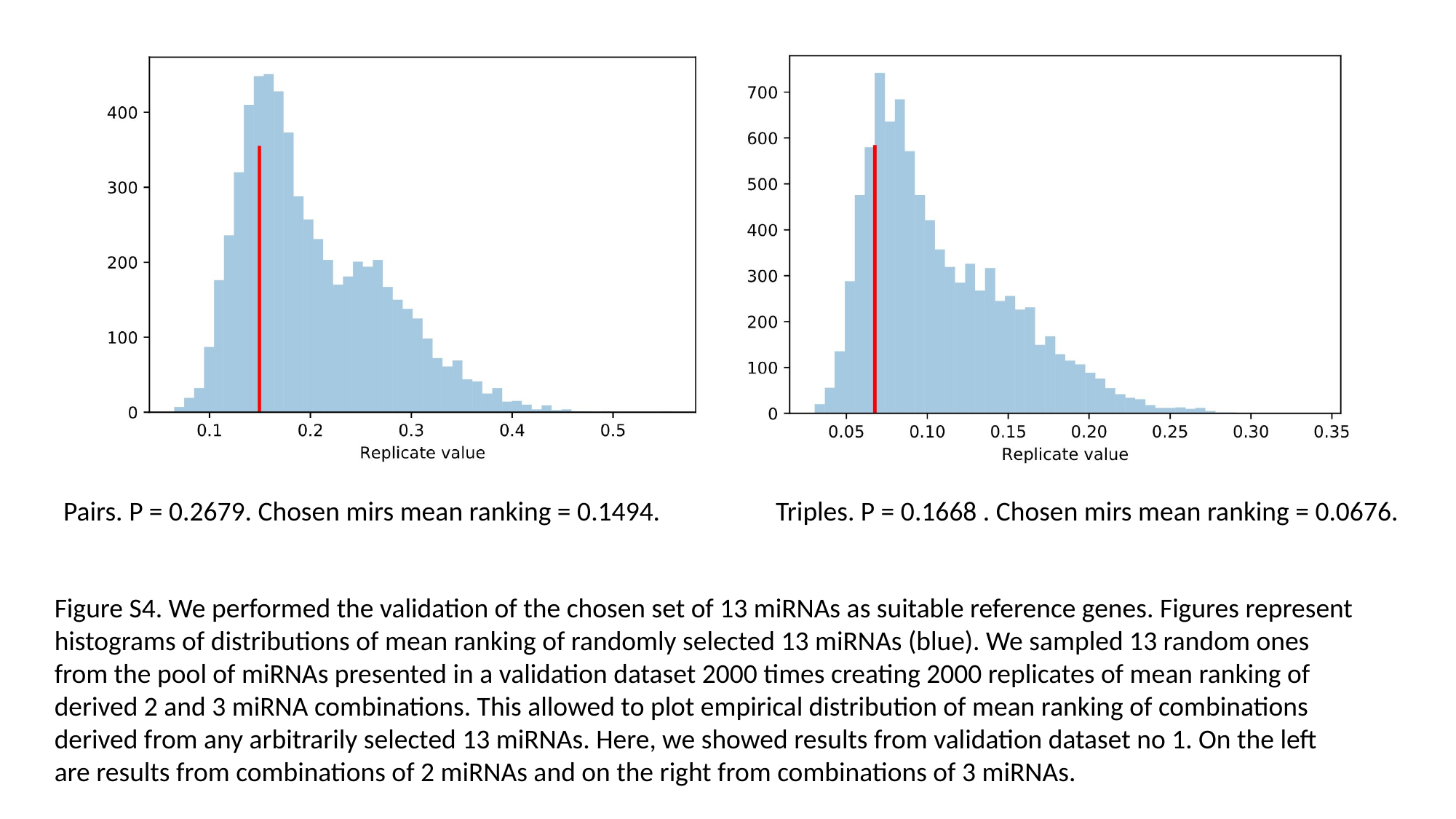

Pairs. P = 0.2679. Chosen mirs mean ranking = 0.1494.
Triples. P = 0.1668 . Chosen mirs mean ranking = 0.0676.
Figure S4. We performed the validation of the chosen set of 13 miRNAs as suitable reference genes. Figures represent histograms of distributions of mean ranking of randomly selected 13 miRNAs (blue). We sampled 13 random ones from the pool of miRNAs presented in a validation dataset 2000 times creating 2000 replicates of mean ranking of derived 2 and 3 miRNA combinations. This allowed to plot empirical distribution of mean ranking of combinations derived from any arbitrarily selected 13 miRNAs. Here, we showed results from validation dataset no 1. On the left are results from combinations of 2 miRNAs and on the right from combinations of 3 miRNAs.

## Slide 5
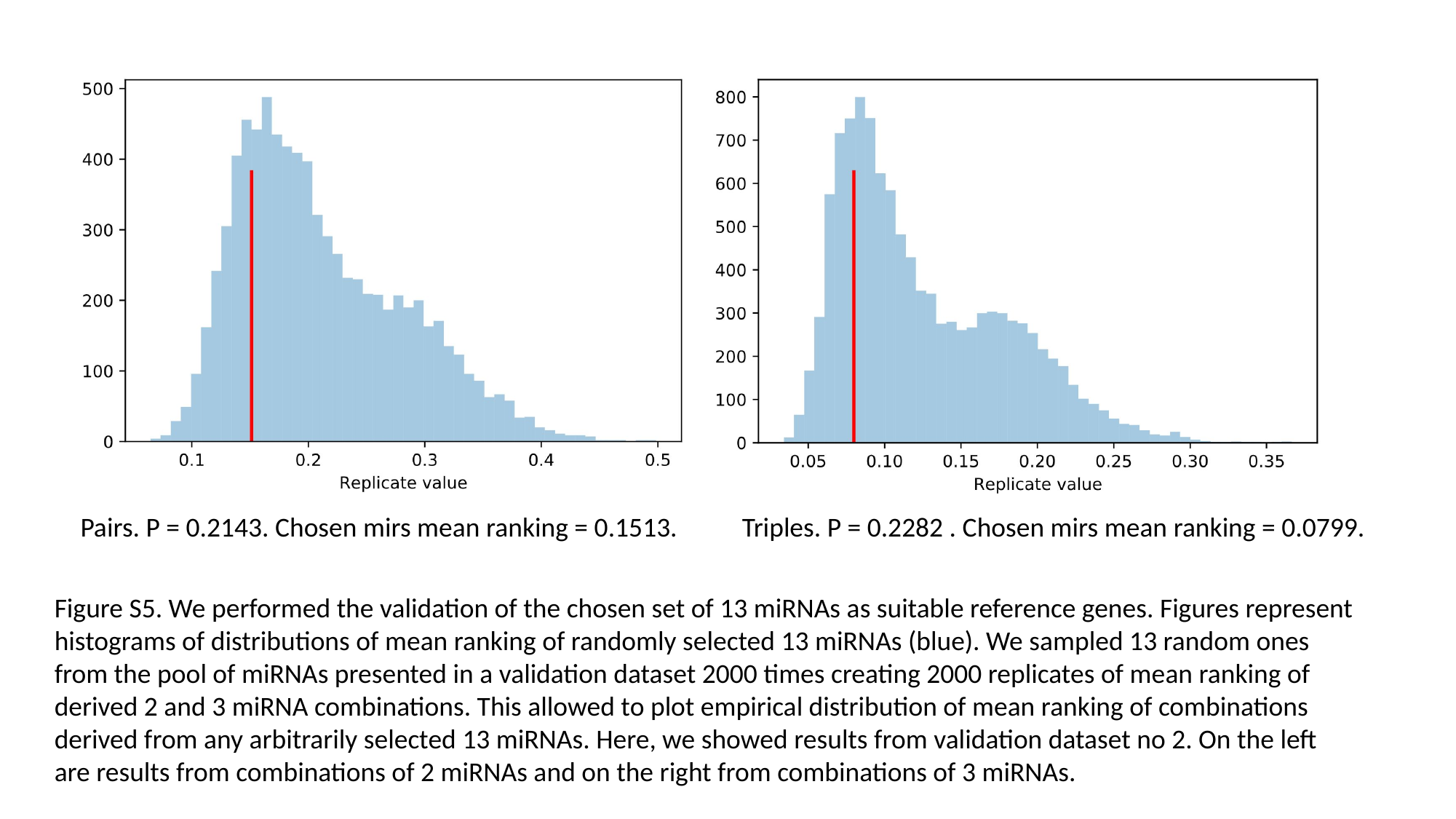

#
Pairs. P = 0.2143. Chosen mirs mean ranking = 0.1513.
Triples. P = 0.2282 . Chosen mirs mean ranking = 0.0799.
Figure S5. We performed the validation of the chosen set of 13 miRNAs as suitable reference genes. Figures represent histograms of distributions of mean ranking of randomly selected 13 miRNAs (blue). We sampled 13 random ones from the pool of miRNAs presented in a validation dataset 2000 times creating 2000 replicates of mean ranking of derived 2 and 3 miRNA combinations. This allowed to plot empirical distribution of mean ranking of combinations derived from any arbitrarily selected 13 miRNAs. Here, we showed results from validation dataset no 2. On the left are results from combinations of 2 miRNAs and on the right from combinations of 3 miRNAs.

## Slide 6
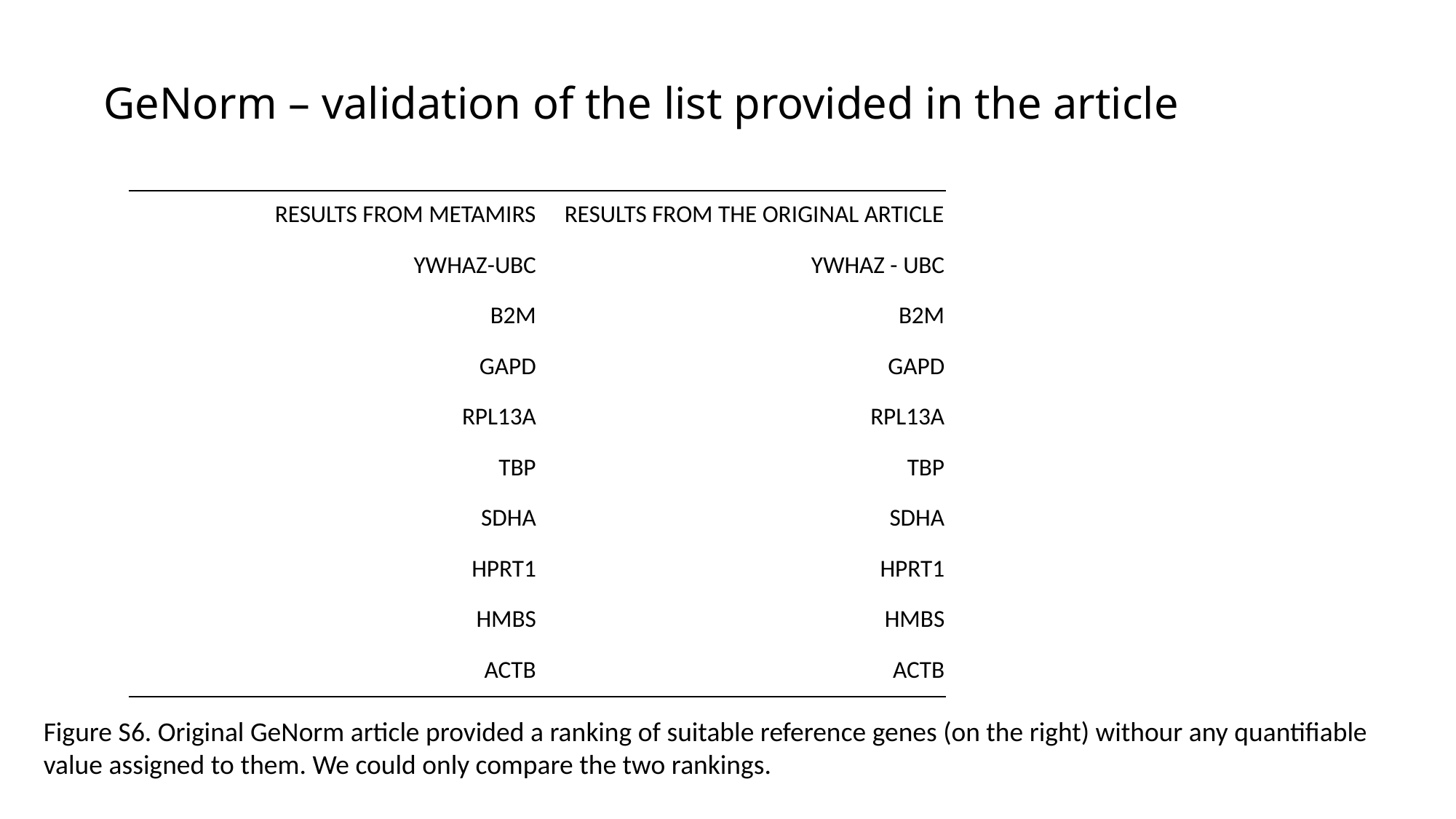

# GeNorm – validation of the list provided in the article
| RESULTS FROM METAMIRS | RESULTS FROM THE ORIGINAL ARTICLE |
| --- | --- |
| YWHAZ-UBC | YWHAZ - UBC |
| B2M | B2M |
| GAPD | GAPD |
| RPL13A | RPL13A |
| TBP | TBP |
| SDHA | SDHA |
| HPRT1 | HPRT1 |
| HMBS | HMBS |
| ACTB | ACTB |
Figure S6. Original GeNorm article provided a ranking of suitable reference genes (on the right) withour any quantifiable value assigned to them. We could only compare the two rankings.
